# Scaling Geriatric and Telemedicine Care for Older Adults in Rural Areas Through Clinical Strategies and Training

**DOI:** 10.1093/geroni/igaa057.2883

**Published:** 2020-12-16

**Authors:** William Hung, Becky Powers, Stuti Dang

## Abstract

Telemedicine, the use of electronic information and communication technologies to deliver care, has grown substantially over the past few years, potentially benefiting older adults who have difficulty accessing and traveling to care locations. Given that providers and interprofessional staff with training in geriatric medicine often practice in urban rather than rural areas, older adults’ access to quality geriatric care is limited. Prior experiences with telemedicine adoption for geriatric team consultation, though limited in scope, were well accepted by older adults and demonstrated benefits such as identifying and meeting care needs for older adults. Bringing geriatric team care to large regions across the country requires further consideration of population needs, local contexts and training and enhancement of an interprofessional workforce to deliver geriatric care through telemedicine. The Veteran healthcare system has been a pioneer in telemedicine care and considers the use of telemedicine necessary for all providers in its system. This symposium aims to discuss approaches to identify and target older adults who may benefit from geriatric consultation, how care delivery is scaled through identifying common approaches and local adaptations, what the important elements are for providers and teams to deliver care effectively for the older adult population, especially those with multiple complex chronic conditions and functional limitations, and considerations for training the next generation of providers to provide care for older adults with complex conditions, particularly in rural areas with limited access.

